# Olfactory navigation in arthropods

**DOI:** 10.1007/s00359-022-01611-9

**Published:** 2023-01-20

**Authors:** Theresa J. Steele, Aaron J. Lanz, Katherine I. Nagel

**Affiliations:** Neuroscience Institute, NYU School of Medicine, 435 E 30th St., New York, NY 10016 USA

**Keywords:** Olfaction, Navigation, Arthropod, Insect, Neural circuits

## Abstract

Using odors to find food and mates is one of the most ancient and highly conserved behaviors. Arthropods from flies to moths to crabs use broadly similar strategies to navigate toward odor sources—such as integrating flow information with odor information, comparing odor concentration across sensors, and integrating odor information over time. Because arthropods share many homologous brain structures—antennal lobes for processing olfactory information, mechanosensors for processing flow, mushroom bodies (or hemi-ellipsoid bodies) for associative learning, and central complexes for navigation, it is likely that these closely related behaviors are mediated by conserved neural circuits. However, differences in the types of odors they seek, the physics of odor dispersal, and the physics of locomotion in water, air, and on substrates mean that these circuits must have adapted to generate a wide diversity of odor-seeking behaviors. In this review, we discuss common strategies and specializations observed in olfactory navigation behavior across arthropods, and review our current knowledge about the neural circuits subserving this behavior. We propose that a comparative study of arthropod nervous systems may provide insight into how a set of basic circuit structures has diversified to generate behavior adapted to different environments.

## Introduction

Odor is a fundamental signal used to locate resources across the animal kingdom. Food sources, be they flowers, fruits, decaying corpses, or live humans, emit chemical signatures that can be transported long distances on wind or water currents and tracked back to their sources to find food. Like food, hospitable sites for egg laying also release chemical cues useful for localization. Finally, many animals release or deposit specific chemicals—pheromones—to enable conspecifics to find important locations in the environment. These include both volatile pheromones released to attract mates, and chemical trails that can be followed by conspecifics to a previously located food source.

Arthropods—whether they’re ants at a picnic or mosquitoes on a summer evening—are famously good at tracking odors. Arthropoda is an enormous and varied phylum, with members found across even the most inhospitable environments. Behavioral studies across arthropod species have highlighted both common and divergent strategies in odor-seeking behavior. The arthropod brain has a highly conserved structure, with dedicated regions for processing odor and flow stimuli, learning temporal associations between scents and food rewards, and representing navigational variables to guide locomotion. However, the nature of the odors that arthropod species encounter and seek, the structure of the odor signals they follow, and the physical constraints of moving are highly variable across ecosystems, driving adaptive changes in sensation and locomotion. In this review, we highlight common features of olfactory navigation behavior that have been observed across arthropod species, and review current knowledge about the neural circuit structures that likely support these behaviors. We further describe key differences in behavior across species and speculate on possible changes in neural circuit structure and function that might underlie them. We argue that a comparative study of olfactory navigation behavior and circuitry across arthropods is likely to yield important insight into how nervous systems have diversified to adapt to different environments, and describe tools and resources that will be required to pursue this research program.

### The complexity of natural odor signals

Odor signals are highly dependent on their environments and differ in both their chemistry and physical structure. One of the largest differences occurs between odor signals in air versus water: volatility determines if a molecule will become gaseous and be dispersed in air, while solubility determines if a molecule may be dispersed in water. Terrestrial plant odors, which are appetitive for many insects, are often molecules such as monoterpenes, aliphatic acids, aromatic alcohols and esters, all of which are relatively insoluble (Raguso 2008; Shields and Hildebrand 2001). Similarly, moth pheromones are generally insoluble fatty alcohols and polyunsaturated hydrocarbons (Zhang and Löfstedt 2015). Finally, CO_2_, a common insect cue released by prey, fermentation, and vegetation, is a volatile gas. In contrast, appetitive odorants for crustaceans are generally molecules like dissolved free amino acids, amines, nucleotides, and peptides, which are readily soluble in water, but not volatile (Derby and Thiel 2014).

**Fig. 1:**
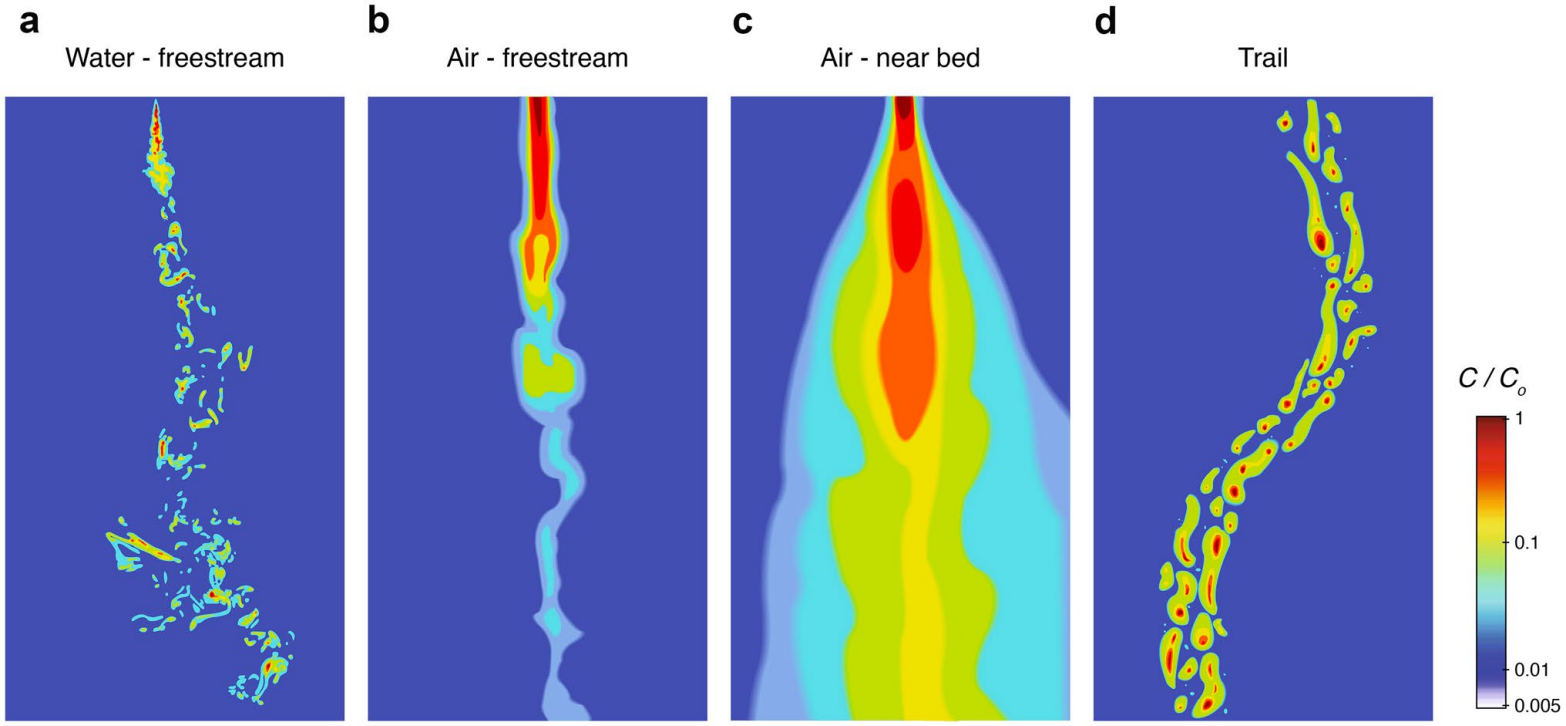
Odor dispersal is driven by features of the environment. **a** Odors in water form filamentous plumes with packets of high odor concentration found downstream of the plume. **b** In air, increased diffusion generates odor plumes with a graded structure. **c** Plumes along substrates, close to a boundary layer, form orderly gradients. **d** Odor trails are deposited on a substrate and can be broken up by weathering. All images depict instantaneous odor structure. Colors represent normalized concentration as a fraction of source concentration. (Adapted from Weissburg 2000, Webster and Weissburg 2009, Connor et al. 2018, Draft et al. 2018)

The physical structure of odor signals also differs between air and water (Fig. 1). Odors that disperse into the environment form structured plumes, which are shaped by both advective transport (i.e., odor motion via flow) and diffusive transport (i.e., odor motion via molecular diffusion, Crimaldi and Koseff 2001; Crimaldi et al. 2002; Connor et al. 2018; Celani et al. 2014). This creates structural differences between plumes in water and air. In water, advection dominates, resulting in filamentous plumes with fairly high odorant concentrations within each filament, even distant from the source (Fig. 1a). By contrast, plumes in air are subject to more diffusive transport, resulting in odor plumes with a more graded structure (Fig. 1b). In both cases, plume structure is shaped by turbulence, which varies with distance from a substrate, or “boundary layer” (Crimaldi et al. 2002). In the layer closest to the surface, flow is slow and odors form gradients that vary minimally over time (Fig. 1c). In contrast, in free stream conditions, odor forms temporally intermittent plumes with more filamentous structure. In a viscous substrate, odor does not form a plume but rather a smoother gradient (Louis et al. 2008). This generates clear differences in the sensory landscapes experienced by organisms moving through fluids (i.e., swimming or flying) compared to those moving on a substrate or within a medium such as larvae.

Features of the landscape can also shape the spatial and temporal structure of odor plumes. As fluid moves over obstructions, it sheds eddies, which can dramatically impact odor dispersal—for example, gaps between odor filament encounters are much longer in forests compared to fields, allowing plumes to meander over greater distances in fields (Murlis et al. 2000). Eddy formation is also a function of the speed of the fluid, and as wind speeds and turbulence vary with time of day and position within a forest canopy, odor plumes become wider-ranging depending on time of day and canopy location (Thistle et al. 2004; DePasquale et al. 2022). The size of eddies is also driven by the viscosity of the fluid, meaning that eddies in air are much larger than those in water (0.1–1 mm in aquatic habitats, 1–10 mm in air).

In contrast to air or water-borne plumes, chemical trails are deposited directly on a substrate (Draft et al. 2018; Jinn et al. 2020) and typically do not fluctuate due to turbulence (Fig. 1d). Nevertheless, such trails can be disrupted by wind or water currents, by odorant evaporation, or by the movement of other animals (Jinn et al. 2020). Trails are often narrow, meaning the animal will experience large odor concentration changes as it moves and searches along the trail. Thus, a need to process dynamically changing odor stimuli is likely common across olfactory environments, although the precise dynamics, and their relationship to animal movement, will be specific to each type of “odor landscape” (Crimaldi et al. 2022).

### Diverse strategies for determining odor source direction and location

**Fig. 2:**
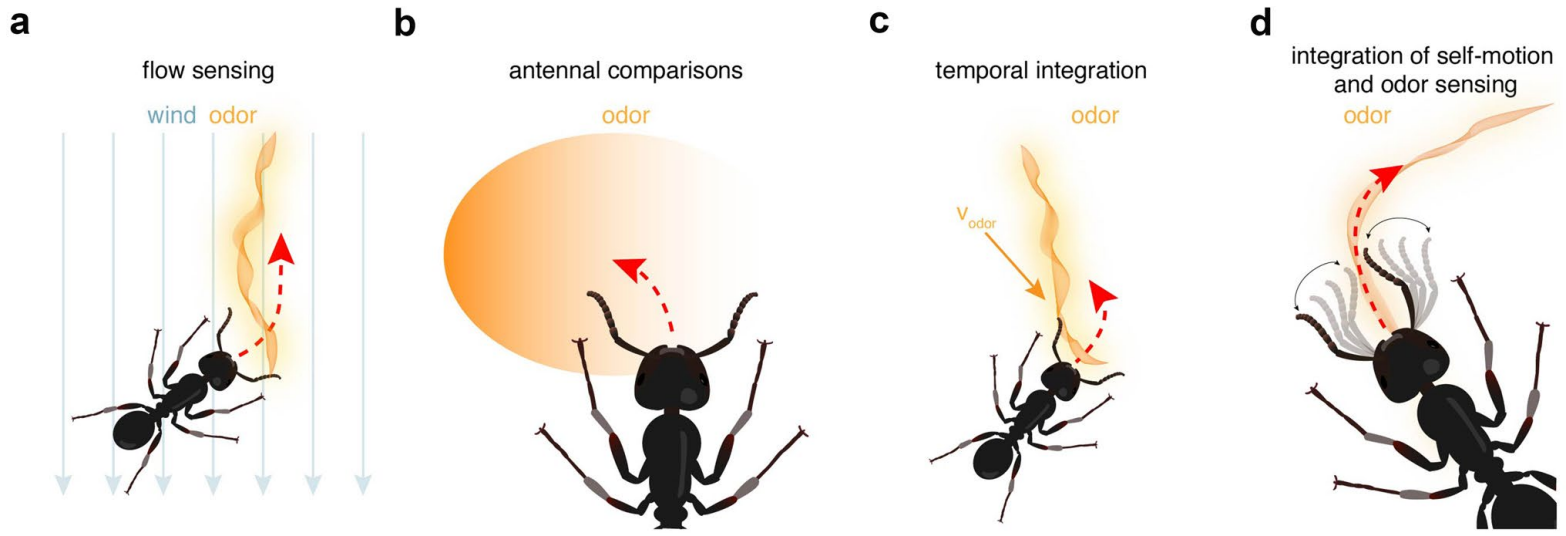
Animals use diverse strategies for odor source localization. **a** Flow taxis is a common strategy for navigating turbulent plumes, as flow direction represents a more reliable signal of source direction than local concentration. **b** Spatial comparisons across antennae facilitate the detection of odor gradients and odor motion based on concentration and timing differences. **c** Integration of plume dynamics over time can be used to infer odor source location based on reliable plume statistics. **d** Many of these strategies imply the use of spatial memory to integrate sensory observations with movement of the antennae or body. For example, an ant using measurements over successive antennal sweeps to follow an odor trail must integrate local measurements of odor with knowledge of antennal motion to identify trail direction. Thus, a variety of cues, requiring different levels of computation, play a role in localizing an odor source

A fundamental challenge in olfactory navigation is that the direction of an odor source is often not clear from instantaneous measurements of odor concentration. As described above, odor signals tend to be highly dynamic and variable in both space and time, meaning that navigators often must integrate instantaneous measurements of odor, or rely on non-olfactory cues, to make reasonable inferences about odor source location. Several strategies such as flow taxis, bilateral sensory comparisons, and integration of measurements over time, appear across many species to facilitate such inference (Fig. 2). Even in environments where odors form orderly gradients, navigators such as larval *Drosophila* are known to employ temporal integration (Gershow et al. 2012; Schulze et al. 2015; Gomez-Marin et al. 2011; Gepner et al. 2015) as well as spatial active search strategies such as head-casting (Gomez-Marin et al 2011) to compensate for shallow or noisy signals (Louis et al. 2008; Gomez-Marin et al 2011).

A widespread strategy for finding the source direction in more turbulent environments is to measure the flow direction (Fig. 2a). As odors are typically transported by wind or water currents, average flow direction represents a more reliable signal of odor source location than instantaneous concentration. Flow taxis has been observed in moths (Kennedy and Marsh 1974), mosquitos (Dekker and Carde 2011), flying and walking *Drosophila* (Budick and Dickinson 2006; Alvarez-Salvado et al. 2018; van Breugel and Dickinson 2014), cockroaches (Willis and Avondet 2005), tsetse flies (Gibson et al 1991), blue crabs (Zimmer-Faust et al. 1995; Page et al. 2011a, 2011b), crayfish (Kozlowski et al. 2003), and lobsters (Moore et al. 1991), among others. Animals standing on a substrate can estimate the flow direction from mechanosensory deflections of their antennae (Alvarez-Salvado et al. 2018; Suver et al. 2019), while free-flying or swimming animals must combine optic flow with mechanosensory information to estimate the direction of ambient wind or water-flow (Kennedy 1940; Rutkowski et al. 2011; van Breugel and Dickinson 2014; van Breugel et al. 2022). This is because in flight and swimming, only changes in flow can be detected through mechanosensation; steady-state flow instead transports the animal itself, resulting in an optic flow signal displaced from the animal’s intended direction of movement (Kennedy 1940; Rutkowski et al. 2011; van Breugel et al. 2022). Flow direction can be measured instantaneously, or can be integrated over time and stored to obtain a more reliable estimate of a noisy variable (Willis and Avondet 2005; Grunbaum and Willis 2015).

Comparisons across sensors are another common strategy for determining odor source direction (Fig. 2b). Walking *Bombyx mori* moths use antennal comparison of odor timing and intensity to set heading (Takasaki et al. 2012), as do crayfish (Kraus-Epley and Moore 2002) and cockroaches (Bell and Tobin 1981, 1982). Blue crabs have been shown to use comparisons across both leg and antennal chemosensors to navigate (Keller et al. 2003; Page et al. 2011a, b). Although fruit flies have very closely spaced antennae, recent work has shown that flies can use cross-antennal comparisons to locate a male producing the pheromone cVA when within 5 mm of the source (Taisz et al. 2022), and that bilateral comparisons contribute to food odor-localization in flies, though they are not required (Duistermars et al. 2009; Wasserman et al. 2012; Louis et al. 2008). A recent study has suggested that odor motion may also be extracted through bilateral sensor comparison, and serve as a cue for predicting the centerline of a plume (Kadakia et al. 2022). Cross-sensor comparisons may also occur along the length of an antenna, such as in the American cockroach (*Periplaneta Americana*). In this species, central neurons exhibit spatial tuning for locations along the antenna (Nishino et al. 2018; Paoli et al. 2020), and the total length of antennae is a greater determinant of successful odor source tracking than the presence of bilateral sensors (Lockey and Willis 2015). Finally, active movements of the antennae or legs can provide additional directional or spatial information, but require the animal to keep track of its movements to interpret the resulting odor signals. For example, ants tracking a chemical trail will sweep their antennae across the trail to determine which direction to go (Draft et al. 2018), and larvae will sweep their heads from side to side, ultimately selecting the direction with the largest concentration gradient (Gomez-Marin et al. 2011). Together these studies point to a range of different computations involving comparisons across sensors on different parts of the body, as well as computations that require integration and storage of sensor position information with odor information.

Although the precise dynamics of odor plumes are chaotic and therefore unpredictable, the statistical properties of odor plumes are reproducible and contain information that animals could use to navigate (Boie et al. 2018; Victor et al. 2019; Crimaldi et al. 2022). Odor plumes in air decrease in concentration with longitudinal distance from the plume source and become more sparse with lateral distance (Crimaldi et al. 2002; Connor et al. 2018). Odor motion preferentially occurs laterally from the plume center (Fig. 2c, Kadakia et al. 2022). Moreover, sources that are separated from one another create plumes that are decorrelated in time, allowing for separation of sources at a distance (Ackels et al. 2021). Evidence from several species suggests that rapid plume fluctuations—which are characteristic of the plume center—can promote more effective upwind navigation (Demir et al. 2020; Baker et al. 1985, 1990). Overall, the insect olfactory system appears to be adapted for high temporal resolution (Szyszka et al. 2014), facilitating the measurement of high odor encounter frequencies. Thus, temporal cues in the odor plume as well as motion signals may help navigating animals locate the plume center.

Many of the strategies described above either require or are aided by different forms of memory. For example, measuring the statistics of odor encounters requires temporal integration (Fig. 2c). Active strategies for measuring concentration gradients, such as head casting and antennal sweeping, require an animal to integrate information about the position of its body or sensors with the timing of odor encounters to extract direction information (Fig. 2d). Additionally, animals may integrate odor encounters with body motion information as they move through the world. Supporting this idea, ants can use learned olfactory cues to walk at a direction offset from the wind direction toward their nest (Steck et al. 2010), and models that incorporate spatial memory have proven effective in allowing artificial agents to navigate towards odor sources (Grunbaum and Willis 2015). Some insects such as bees form explicit spatial memories of where food sources were encountered and can navigate between them using novel routes, suggesting a form of vector memory (Le Moël et al. 2019). Together these observations point to a role for spatial memory processes in olfactory navigation, as well as the integration of sensory information with self-motion information to generate representations of the olfactory environment. Thus, different forms of memory, ranging from simple integration over time, to sensory-motor integration, to vector memory may play a role in olfactory navigation in diverse species.

Although classical work on olfactory navigation sought to isolate single cues or algorithms used to find an odor source, more recent work has emphasized the combination of multiple cues to more robustly navigate in complex environments (Alvarez Salvado et al. 2018; Jayaram et al. 2022). Experimental approaches for measuring the integration of various cues, and computational models for how these cues are integrated are likely to prove fruitful in understanding how arthropods are able to find food and mates reliably despite the complexity and ambiguity of natural odor signals. A deeper understanding of the neural circuit structures underlying navigation, as discussed below, will allow us to understand how diverse direction cues are effectively integrated for robust navigation.

### Common features of odor-seeking behavior and species-specific variations

**Fig. 3:**
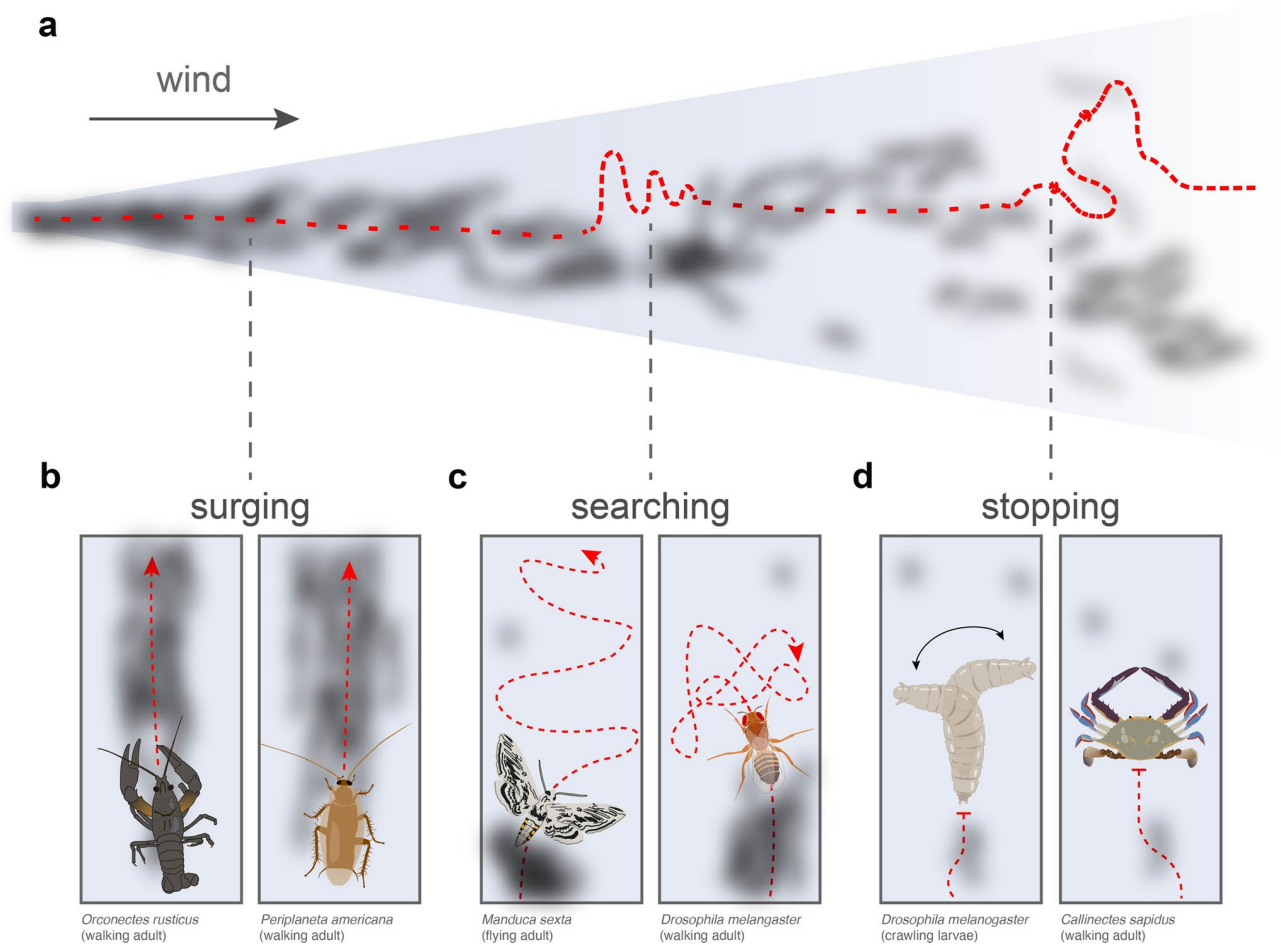
High-level components of olfactory navigation are conserved across species. **a** Arthropods use a combination of surging, searching, and stopping to navigate to an odor source. **b** Surging consists of relatively straight, directional movements, that generally persist while contacts with odor are frequent. In different species, surging may be driven by different sensory signals (e.g., pulsed or constant plumes), and occur with different speeds due to locomotor differences. **c** Searching, or “casting”, follows loss of the plume and consists of increases in turning and path curvature. As with surging, the dynamics of searching vary between species—flying *Manduca* (left) perform the highly stereotyped casts typical of pheromone tracking in moths, while walking *Drosophila* (right) perform irregular searching on loss of odor. **d** Stopping may occur when odor contacts are infrequent, and can allow for evidence accumulation via active search (*Drosophila* larval head casting, left) or through observations over time (*Callinectes* adult, right)

Although arthropods use a wide array of locomotor strategies, from peristaltic waves in larvae (Clark et al. 2018), to walking in adult insects (Büschges et al. 2008), to flight (Sane 2003) and swimming (Zhang et al. 2014), classic behavioral work in moths, fruit flies, mosquitoes, and crabs has identified core olfactory navigation behaviors found across many arthropods. These include ‘*surging*,’ in which an organism responds to an appetitive odor by navigating in a defined direction; ‘*searching’,* or casting behavior driven by the loss of odor; and ‘*stopping*’, or pausing to obtain more information about the odor source direction (Fig. 3). These high-level behavioral components are observed in many arthropods despite differences in the mode of locomotion. However, details of how these components are utilized vary across organisms.

One of the most common behavioral strategies arthropods employ is surging. Surging consists of movement that is typically straighter and faster than baseline locomotion, in a defined direction (Fig. 3b). In many cases, the direction of the surge is defined by flow, as discussed above (David et al. 1983; van Breugel et al. 2014; Alvarez-Salvado et al. 2018; Demir et al. 2020). In other cases, the surge direction is determined from cross-antennal comparisons (Takasaki et al. 2012; Draft et al. 2018). Additionally, while surging behaviors in most arthropods are informed by flow sensing, in larval *Drosophila*, who occupy a very low-flow environment, surging behavior is also seen in response to concentration gradients (Gershow et al. 2012). Surging is nearly always evoked by the presence of attractive odor, but the duration of surges and the stimulus dynamics required to evoke surging differ across species. In moths, surges are transient and pulsed pheromone is required to maintain upwind progress (Baker et al. 1985, 1990). In contrast, in both flying and walking *Drosophila*, sustained upwind progress occurs as long as an attractive odor (apple cider vinegar or ethanol) is present. The dynamics required to elicit upwind progress can depend on odor as well as species. In flying *A. aegypti* mosquitoes, CO_2_ will only evoke strong upwind flight when presented as a filamentous plume, while skin odors can evoke the same behavior when presented as a broad, homogenous plume (Dekker and Cardé 2011; Dekker et al. 2005). A similar difference has recently been observed in Drosophila, where vinegar produces upwind walking when presented continuously, while CO2 must be pulsed to evoke upwind movement (Zocchi et al. 2022). Thus, the same behavior may be driven by different odor dynamics across species and even odors.

Upon losing contact with odor, many arthropods exhibit some form of searching to facilitate finding the lost plume. A common feature of these behaviors is a change in the statistics of turning relative to both baseline locomotion and the straighter surges; although, as with surging, the details of these turning behaviors differ across species and locomotor modes (Fig. 3c). For example, many insects perform ‘casts’, crosswind motions with or without upwind displacement (Kennedy et al. 1974; van Bruegel et al. 2014). In flying moths, casts consist of extremely regular side to side motions in the plane parallel to the ground (Kuenen and Carde 1994; David et al. 1983). In some moth species, casting occurs even in homogenous clouds of odor (Kennedy et al. 1981), while in others, casting occurs in response to loss of the plume (Vickers and Baker 1994; Kennedy 1983; Takasaki et al. 2012). Like moths, casts in flying mosquitoes occur at stereotyped angles and velocity relative to the wind, and are initiated by loss of contact with the odor plume (Dekker and Carde 2011). Flying *Drosophila* exhibit crosswind casts of variable speed and duration which are stronger at higher odorant concentrations and weaken over time with successive odor encounters (Van Bruegel and Dickinson 2014; Pang et al. 2018). However, walking flies instead show a non-directional local search upon odor loss (Alvarez-Salvado et al. 2018). Walking cockroaches (*P.americana*) and silk moths (*B.mori*) also exhibit cast-like turning upon loss of odor, but these casts are much more variable in their timing and structure than those of moths (Willis and Avondet 2005; Takasaki et al. 2012). Casting behavior is also observed during trail following upon loss of the odor trail in both ants (Draft et al. 2018) and copepods (Weissburg et al. 1998) but is notably absent from the olfactory behavior of many walking crustaceans such as lobsters (Moore et al 1991), blue crabs (Page et al. 2011a, b), and crayfish (Kozlowski et al. 2003).

Stopping or pausing is an often-overlooked component of navigation. Stopping, which consists of the cessation of forward motion, has generally been considered an endpoint of navigation (i.e., abandonment of the plume on loss of odor), rather than a bona fide navigation strategy. However, evidence from walking arthropods—insect and crustacean—suggests stopping plays an integral role in evidence accumulation (Fig. 3d). Crawling *Drosophila* use stops to perform head casts, which actively sample the environment to correct course (Gomez-Marin et al. 2011). Ants also pause to perform additional antennal sweeps of a trail (Draft et al. 2018), suggesting that pausing is a common strategy that allows animals to obtain additional information about the likely direction of the odor source. Observations on pausing during plume tracking support this hypothesis. Blue crabs exhibit increased stop frequency as the turbulence of an odor plume increases (Weissburg and Zimmer-Faust 1994); similarly, crayfish stop with increasing frequency as odor pulse rate decreases (Kozlowski et al. 2003). Recent work in walking *Drosophila* has shown that stop frequency evolves dynamically over time—odor filament encounters produce a transient decrease in the rate of stopping, which eventually decays back to baseline (Demir et al. 2020). These findings suggest that across species, the statistics of stopping are driven by an organism’s ability to integrate information about its environment (Rigolli et al. 2022).

### Ecological mechanisms underlying variations in olfactory navigation behavior

This survey of olfactory navigation behaviors suggests that a set of common high-level behaviors—surging, casting, and pausing—are evoked by different stimuli and stimulus dynamics across species. How might we understand these behavioral motifs as a product of the olfactory environments encountered by an animal? Weissburg (2000) proposed a unifying framework to describe how the reliability of olfactory signals in different environments might shape navigation strategies. This model proposes two factors impacting navigation strategies—a temporal integration factor, describing an animal’s capacity to compute time-averaged plume statistics, and a spatial integration factor characterizing the ability to instantaneously measure the statistics of a plume across space. These factors are influenced both by the dynamics of the environment (the width of an odor plume and the size of the smallest eddies in the plume, which are both related to Reynolds number) and by an organism’s own anatomy and behavior (e.g., span of antennae and speed of locomotion). For example, flying moths with high movement speeds and narrowly spaced antennae would have low spatial and temporal integration capacities, suggesting that wind direction may be a more salient signal of odor source than any features of the plume itself. On the other hand, walking lobsters have a low sampling rate and therefore less effective temporal integration, but much larger capacity for spatial integration due to the length of their antennae, making strategies dependent on bilateral sensing or other comparisons across space more salient.

New tools for detailed, high throughput quantification of olfactory behaviors may allow scientists to test and expand on this intriguing hypothesis. In particular, the literature reviewed above suggests that animals adopt active strategies to increase their capacity to collect and integrate information about a plume. Modeling approaches suggest that insect-like searching behaviors arise as an optimal solution to navigating a turbulent plume through a Bayesian inference process (Rigolli et al. 2022). Capitalizing on technological innovations to capture the fine details of navigation in arthropods adapted to a range of environments may reveal guiding principles for how these high-level behavioral strategies have evolved to match the statistics of odor plumes across different environments.

### Neural circuits for olfactory navigation

**Fig. 4:**
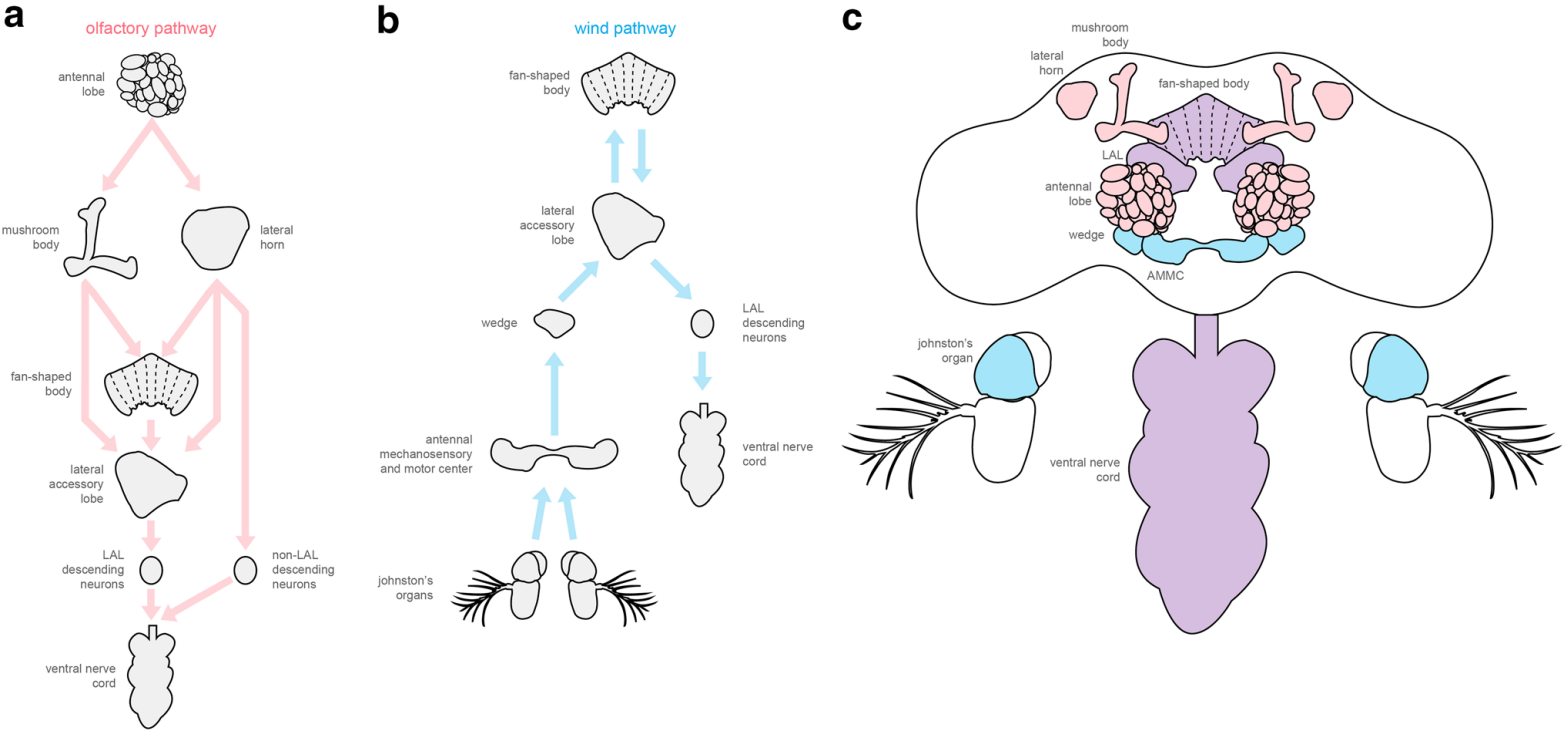
Odor and wind processing pathways in *Drosophila*. **a** Glomeruli in the antennal lobe integrate signals from antennal olfactory sensory neurons, and pass this information to two associative centers, the mushroom body and the lateral horn, which compute learned and innate valence, respectively. A subset of mushroom body and lateral horn outputs are combined with wind information in the fan-shaped body of the central complex to generate navigational signals. These signals are passed to the premotor lateral accessory lobe and ultimately drive behavior through motor neurons in the ventral nerve cord. Other mushroom body and lateral horn outputs bypass the central complex to drive behavior through direct pathways to the LAL or other descending inputs to the ventral nerve cord. **b** Stretch receptive Johnston’s organ neurons (JONs) that project to the antennal mechanosensory and motor center (AMMC) respond to displacement of the antennae due to wind. This information is passed to the wedge, where inputs from the two antennae are integrated to generate a representation of wind direction. Other WED neuron carry displacement information to the LAL and then to central complex. In the fan-shaped body of the central complex, wind direction and odor value information is integrated. This information is thought to descend via the LAL to drive activity in the VNC. **c** Anatomically distinct circuits for odor (pink) and wind (blue) sensing converge in navigation centers (purple, detailed in Fig. 5) to drive movement toward an odor source

Why are common behavioral motifs observed across organisms with highly divergent body plans, and how do species-specific variations in olfactory navigation behavior arise? Efforts to address this question will require a detailed understanding of the neural circuitry that gives rise to olfactory navigation behavior. While decades of work have investigated olfactory and navigational circuits across a range of insects (Heinze and Homberg 2008; Namiki and Kanzaki 2014; Martin et al. 2015) and crustaceans (Ache and Derby 1985), major breakthroughs have occurred in recent years due to the power of *Drosophila* as a model for genetic dissection of neural circuits (Guo et al. 2019). In the following sections, we discuss what is currently known about neural circuits for detecting and processing odor and flow information, synthesizing these information streams to generate navigational commands, and translating these commands into locomotor behavior (Fig. 4). Our discussion will focus on recent work using *Drosophila*, but include important contributions from other arthropod models. Finally, we will consider what is known about the evolution and development of these circuit structures, and describe new tools and technologies that will enable comparisons of circuit structure and function across species. We hypothesize that such a comparative approach will help reveal which features of circuit organization are essential and which vary across species.

### Olfactory circuits

Arthropod olfaction is initiated by a large set of olfactory receptor neurons (ORNs) in the antennae (Fig. 4a). ORNs express olfactory receptors that allow them to transduce odors into electrical signals (Vosshall et al. 1999, 2000; De Bruyne et al. 1999, 2001; Hallem et al. 2004; Hallem and Carlson 2006; Silbering et al. 2011). Arthropods use many receptor families as olfactory receptors, most notably the olfactory receptor (ORs) family, which are found only in insects (Vosshall et al. 2000; Hallem and Carlson 2006), and the ionotropic receptors (IRs), which are found more broadly in all protostomes, including arthropods (Croset et al. 2010; Rytz et al. 2013; Corey et al. 2013; Kozma et al. 2020a,b). Both of these receptors function as ion channels (Sato et al. 2008; Wicher et al. 2008; Benton et al. 2009), and require co-expression of a pore-forming co-receptor (orco for ORs, and IR8a or IR25a for IRs), and a more variable “tuning” receptor that confers odorant-specificity (Larsson et al. 2004; Benton et al. 2006; Abuin et al. 2011; Del Marmol and Ruta 2021). As in many sensory systems, ORNs show strong selectivity for specific odors at low concentrations, and become less selective at higher concentrations (de Bruyne et al. 1999, 2001; Hallem et al. 2006). Canonical accounts found that each olfactory receptor neuron expressed a single tuning OR, accounting for the differences in odor tuning across ORN types (Vosshall et al. 2000; Couto et al. 2005; Hallem et al. 2004). However, recent studies in mosquitoes (Herre et al. 2022) as well as flies (Task et al. 2022) have called this model into question, suggesting much greater overlap of receptor expression in individual ORNs. Additional receptor families, such as ppk channels, TRP channels, and gustatory receptors (GRs) have also been shown to act as chemoreceptors in subsets of arthropod neurons (Joseph and Carlson 2015). Activation of ORNs has been shown to produce navigational phenotypes in both larval and adult flies (Schulze et al. 2015; Gepner et al. 2015; Matheson et al. 2022), with different patterns of ORN activation preferentially driving different patterns of locomotor output (Jung et al. 2015; Matheson et al. 2022).

In most arthropod olfactory systems, axons of ORNs that express the same receptor type converge on a smaller number of projection neurons (PNs) in structures known as glomeruli (Couto et al. 2005; Loesel et al. 2013). Some olfactory specialists, such as moths, exhibit “macroglomeruli” for pheromone odors that may play a role in boosting signal strength for these ethologically meaningful odors, as well as in detecting specific pheromone blends (Rospars and Hildebrand 2000; Belmabrouk et al. 2011). Olfactory coding is further refined by local circuitry—mostly composed of inhibitory interneurons—that performs functions such as gain control (Root et al. 2008; Olsen et al. 2008) and decorrelation of olfactory receptor input (Olsen et al. 2010). Local circuitry can also contribute to spatial comparisons. Although fly ORNs project to both hemispheres, these synapses have asymmetric weights, resulting in encoding of cross-antenna intensity differences at the PN level (Gaudry et al. 2013). A recent study identified a single inhibitory interneuron that amplifies cross-antennal differences to produce a spatial code for the direction of the pheromone cVA at short distances (Taisz et al. 2022). Antennal lobe interneurons are highly variable in their morphology even across individuals in one species (Chou et al. 2010) and express a wide variety of peptides that can modulate olfactory processing, for example in response to hunger state (Ignell et al. 2009; Root et al. 2011; Ko et al. 2015; Lizbinski et al. 2018); variations in inhibitory connectivity are one site where differences in the spatial or temporal selectivity of navigation behavior across species might arise.

From the primary olfactory lobe (the antennal lobe in insects), odor information is carried to higher order olfactory areas by a variety of projection neurons (PNs). Uniglomerular PNs carry information from a single glomerulus—reflecting mostly input from one receptor type—while multi-glomerular PNs carry information from groups of glomeruli (Berck et al. 2016; Liang et al. 2013; Vogt et al. 2021). While projection neurons target a number of regions (such as the posterior lateral protocerebrum and anterior ventro-lateral protocerebrum) in different species, (Tanaka et al. 2012; Loesel et al. 2013), the primary targets of olfactory information in insects are the mushroom body and the lateral horn (Bates et al. 2020; Schlegel et al. 2021) (Fig. 4a).

The mushroom body (MB) is an associative structure implicated in olfactory learning. Many years of experiments support this role, starting from the observation that chemical ablation of the mushroom body abolishes learned odor aversion, but not innate aversion (de Belle and Heisenberg 1994). Olfactory projection neurons synapse on to a subset of the input neurons of the mushroom body, which are known as Kenyon Cells (KCs). KCs are the most numerous cell type in the insect brain (2000–50,000 per brain in flies, Caron et al. 2013, 180,000 per brain in bees, Sachse and Gallizia 2006), and are thought to decorrelate and sparsify the representation of odors to facilitate odor discrimination and identification (Turner et al. 2008; Honegger et al. 2011). Sparsification is achieved in part through a large inhibitory neuron, known as APL, that innervates most of the mushroom body (Papadopoulou et al. 2011). KCs make synaptic connections onto a much smaller set of mushroom body output neurons (MBONs) in a highly structured series of compartments within the mushroom body (Aso et al. 2014a, b; Owald et al. 2015). Each compartment is innervated by its own dopaminergic neuron (DANs), which controls plasticity of the KC— > MBON synapse (Liu et al. 2012; Hige et al. 2015; Cohn et al. 2015; Aso et al. 2016). DANs encode a variety of innate valence cues such as sweet taste, sugar input, shock, and odors, as well as features of locomotion (Cohn et al. 2015; May et al. 2020; Siju et al. 2020; Vrontou et al. 2021; Zolin et al. 2021; Kato et al. 2022). They are therefore though to function as the unconditioned stimulus in classical associative plasticity.

MBONs themselves are thought to link the valence of an odor to an appropriate motor output (Strube-Bloss et al. 2011; Owald et al. 2015). Activation of one set of MBONs has been shown to promote attraction, while another set promotes aversion (Aso et al. 2014b). Several MBONs have been implicated in odor-guided food search on different timescales (Tsao et al. 2018; Sayin et al. 2019). One MBON, labeled by MB077B, shows activity that correlates with upwind running (Handler et al. 2019). More recently, different MBONs were shown to promote upwind/downwind movement or path straightening (Matheson et al. 2022), which might suggest an involvement in olfactory or visual navigation, respectively. Intriguingly, activation of certain MBON groups—such as the alpha lobe cluster MB052B—can produce multiple components of olfactory navigation behavior, including upwind running during stimulation, and search behavior at stimulus offset, suggesting that the MB output encodes high-level behavioral “suites” that promote attraction or aversion. At a minimum, this architecture enables arthropods to avoid or approach odors based on previous experience of rewards and punishments. However, recurrent connections between mushroom body neurons may enable more complicated forms of learning, such as reward prediction-based learning, extinction, second-order conditioning, and context-dependent conditioning (Eschbach et al. 2020). KC— > MBON synaptic plasticity also depends critically on the timing of KC activity relative to DAN activity (Gerber et al. 2014; Handler et al. 2019; Devineni et al. 2021), and different MBON compartments appear to encode memories on different timescales (Hige et al. 2015; Aso and Rubin 2016). Thus, the mushroom body has been proposed to act as an “incentive circuit” that integrates internal state information and odors over multiple timescales to promote goal-directed behavior (Gkanias et al. 2022). Variations in MBON connectivity, neuromodulation, or plasticity rules are another likely site where species-specific differences in the timing and duration of olfactory behaviors might arise.

Olfactory projection neurons also target the lateral horn (LH). Thought to perform innate olfactory processing, the lateral horn is a much more complex structure than the mushroom body, with 496 morphologically identified cell types in *Drosophila,* and complex forms of connectivity (Schlegel et al. 2021). Broadly, recordings from lateral horn neurons support the idea that it clusters odors according to valence (Strutz et al. 2014; Das Chakraborty et al. 2022). Lateral horn odor responses are thought to be more stereotyped than those of mushroom body output neurons (Fisek and Wilson 2014; Jeanne et al. 2018; Frechter et al. 2019; Kohl et al. 2013). In contrast to the mushroom body, only a small number of lateral horn outputs produce identifiable behavioral outputs (Dolan et al. 2019). In particular, one cluster of lateral horn outputs, called LHAD1b2, has been shown to produce approach behavior (Dolan et al. 2019) as well as upwind navigation (Matheson et al. 2022), and is required for some forms of odor attraction (Boehm et al. 2022), while a second cluster called aSP-g controls female receptivity during courtship (Taisz et al. 2022). The AD1b2 cluster of LH output neurons also receives axo-axonal input from mushroom body output neurons (Dolan et al. 2019; Schlegel et al. 2021), suggesting that these two pathways interact to shape behavior. Like the alpha lobe MBON cluster, activation of AD1b2 neurons produces an entire “suite” of behaviors related to attraction, including upwind navigation during stimulation, and search behavior triggered by the loss of stimulation (Matheson et al. 2022). In both the lateral horn and the mushroom body, neurons that produce navigational phenotypes do not show responses dependent on wind direction (Matheson et al. 2022), suggesting that the outputs of these regions represents an odor value signal that is translated into navigational and locomotor commands by downstream circuitry.

### Flow-direction circuits

As described above, a key component of olfactory navigation in many species is the use of air or water-flow cues to provide a directional cue for navigation. In many terrestrial arthropods, such as flies (Álvarez-Salvado et al. 2018; Suver et al. 2019), ants (Wolf and Wehner 2000), and cockroaches (Bell and Kramer 1979), wind direction is sensed by the antennae. Wind from different directions differentially displaces the two antennae, generating a code for wind direction that can be read out by central neurons (Yorozu et al. 2009; Suver et al. 2019; Okubo et al. 2020). These displacements are detected by a chordotonal organ known as Johnson’s organ, which is composed of an array of stretch receptors arranged in bowl-like shape around the joint to detect displacement (Gopfert and Robert 2002; Kamikouchi et al. 2006). Primary stretch receptor neurons, known as Johnson’s organ neurons (JONs), are tuned for both direction and dynamics, and provide wind direction information to a region known as the antennal mechanosensory and motor center (AMMC, Kamikouchi et al. 2009; Matsuo et al. 2014). Stabilizing the antennae abolishes orientation to wind in these species, demonstrating that these mechanosensory signals are the only source of wind direction information for wind-guided navigation (Bell and Kramer 1979; Wolf and Wehner 2000; Alvarez-Salvado et al. 2018).

In flies, several components of the central circuitry that processes wind direction information has recently been elucidated (Fig. 4b). From the AMMC, a set of projection neurons carry information to the nearby wedge (WED, Kamikouchi et al. 2009; Matsuo et al. 2014). Recorded AMMC projection neurons respond primarily to deflections of the ipsilateral antenna. In contrast, the WED contains neurons that encode the difference between antennal deflections (Patella and Wilson 2018; Suver et al. 2019). From the WED, wind direction information is sent both to the antler (ANT, Suver et al. 2019) and to the lateral accessory lobe (LAL, Okubo et al. 2020). Two paths from the LAL carry wind direction information to the fan-shaped body, a part of the central complex (described in detail below). One path carries wind information to the ellipsoid body compass (Okubo et al. 2020), while a parallel path carries wind information to columnar neurons of the fan-shaped body (Currier et al. 2020). These two wind inputs to the central complex appear to have different formats. In the ellipsoid body, neurons represent wind direction as a “compass” with different neurons responding to different directions of wind (Okubo et al. 2020). In contrast, fan-shaped body inputs encode wind direction as the activity of a set of orthogonal “basis vectors,” located at ± 45° to the fly’s midline (Currier et al. 2020). How these two representations function together in wind-guided navigation is not yet clear.

A second group of terrestrial arthropods, including crickets, locusts, cockroaches, and mantis (Jacobs et al. 2008; Boyan and Ball 1990), detect wind using a large collection of hair follicles on two structures on the abdomen known as cerci. Each cercus contains between 750 and 2000 mechanosensory hairs (Jacobs et al. 2008; Boublil et al. 2021), each tuned to specific wind directions and frequencies. Sensory neurons innervating these hairs project into the terminal abdominal ganglion to generate a spatial map of horizontal air direction. Aquatic arthropods, such as crayfish (Tautz et al. 1981; Masters et al. 1982) and lobster (Vedel and Clarac 1976; Laverack 1964), detect water currents using two types of hairs distributed along the antennae. The first type of hair responds to motion in the surrounding water (e.g., by a predator), while the second responds to bending of the antennae due to water flow (Tautz et al. 1981; Masters et al. 1982). The brain circuits that process flow signals from these mechanoreceptors are currently unknown. In the future, it will be interesting to investigate whether the various structures that detect flow information in different arthropods send convergent information to highly conserved structures such as the central complex.

### Navigation circuits

The central complex is an ancient structure thought to form the navigation center of the arthropod brain, and has recently been implicated in olfactory navigation (Matheson et al. 2022, Fig. 5). Early studies of mutant flies with disrupted central complex morphology suggest a role for this structure in visual navigation and control of locomotion (Strauss and Heisenberg 1993). The discovery of a map of sky polarization within the central complex of locusts solidified its role in visually guided navigation (Heinze and Homberg 2007). In recent years, functional studies from genetically identified neurons in *Drosophila*, and connectomic reconstruction of this structure in both flies (Hulse et al. 2021) and bees (Sayre et al. 2021) have provided new insights into its organization and function. A key feature of the central complex is a global heading signal caried by the compass neurons of the ellipsoid body (EB) (Seelig and Jayaraman 2015). This signal can be influenced by visual cues (Omoto et al. 2017; Sun et al. 2017), and mechano-sensory cues about wind direction (Okubo et al. 2020), both of which are carried by different types of ring neurons (Hulse et al. 2021), as well as by self-motion cues carried by neurons known as PENs (Green and Maimon 2017; Turner-Evans et al. 2017). This heading signal is thought to be maintained by recurrent connections within the ellipsoid body that form a ring attractor (Kim et al. 2017), and is propagated to the downstream fan-shaped body through ∆7 neurons of the protocerebral bridge (PB, Lyu et al. 2022; Hulse et al. 2021).

**Fig. 5:**
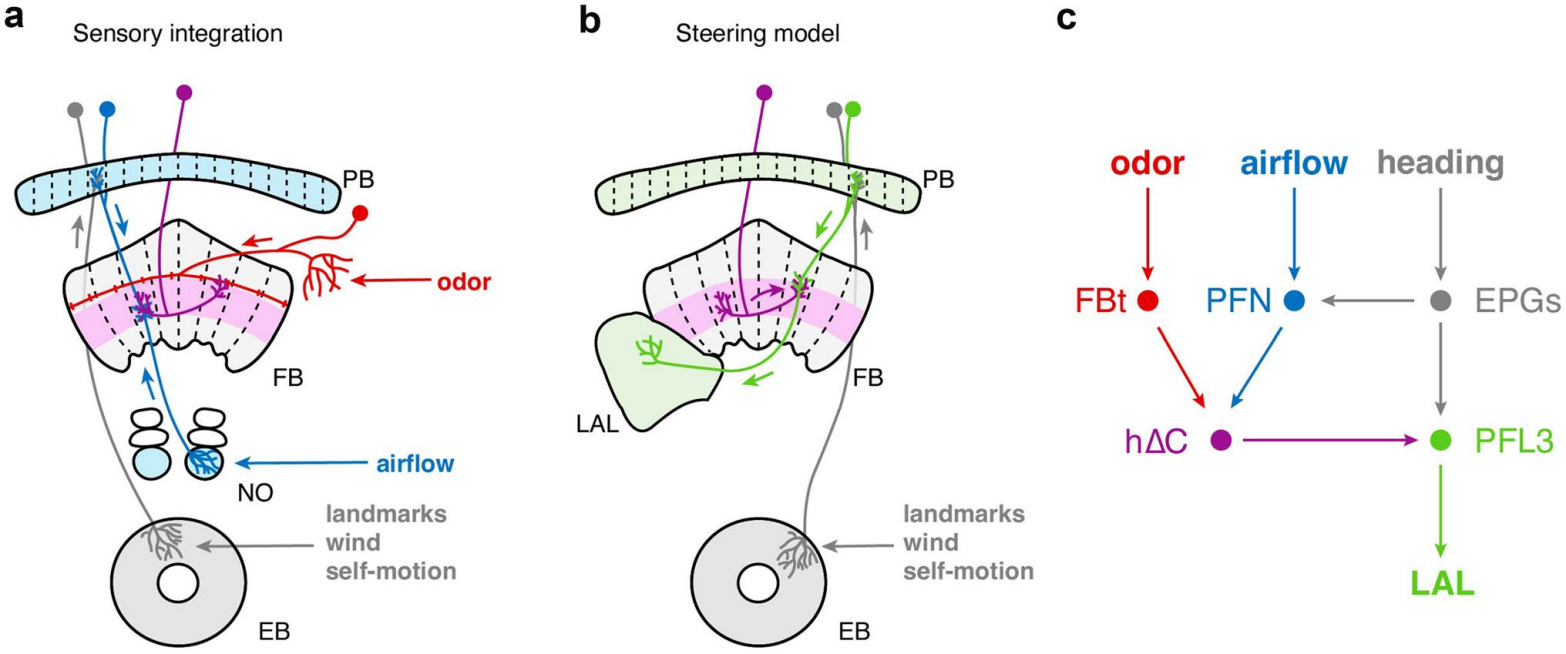
Olfactory navigation circuits within the central complex. The fan-shaped body has been proposed to integrate odor and wind direction signals to generate a goal-direction signal for navigation. **a** Compass neurons (EPGs, gray) carry a multimodal representation of heading and provide input to the fan-shaped body (FB) through the protocerebral bridge (PB). Columnar inputs to the fan-shaped body (PFNs, blue) receive heading input from the PB and airflow information from the noduli (NO). Tangential inputs to the fan-shaped body (red) carry non-directional odor information. Local neurons (h∆C, purple) receive input from both columnar neurons and tangential neurons and show odor-gated wind-direction tuned signals. **b** PFL3 neurons (green) receive phase-shifted heading information from the PB and indirect input from local neurons. They are proposed to control steering through their outputs to the LAL. **c** FB circuits integrate odor, airflow, and heading representation to direct navigation. (Adapted from Hulse et al. 2021; Lyu et al. 2022; Lu et al. 2022; Currier et al. 2020; Matheson et al. 2022.)

The fan-shaped body (FB) is a highly conserved region of the central complex and may play a role in setting navigational goals. The fan-shaped body receives two main types of inputs: columnar neurons (known as PFNs) and tangential neurons (FBt, Fig. 5a). Columnar neurons have been shown to encode spatial direction cues such as wind direction (Currier et al. 2020), optic flow (Stone et al. 2017; Lyu et al. 2022), and self-motion (Lu et al. 2022). Current data suggest that columnar neurons receive sensory inputs from their projections in the noduli (NO), and a heading input through the protocerebral bridge (Lyu et al. 2022; Lu et al. 2022; Currier et al. 2020). These two inputs allow columnar neurons to encode information as vectors, where the angle is given by the bump inherited from the compass, and the magnitude is given by the sensory input (Lyu et al. 2022; Lu et al. 2022; Hulse et al. 2021). In contrast, tangential neurons appear to mostly encode non-spatial “context” cues such as odor (Matheson et al. 2022), tastants (Sareen et al. 2021), and sleep state information (Donlea et al. 2014). Thus, an appealing model is that the architecture of the fan-shaped body allows for non-spatial context cues (encoded by tangential neurons) to select spatial and navigational trajectories encoded by columnar inputs (Honkanen et al. 2019; Hulse et al. 2021; Matheson et al 2022).

Columnar and tangential neurons are connected by a set of local or pontine neurons. One of these groups, called hΔC, was recently shown to encode an odor-gated wind direction signal (Matheson et al. 2022). Silencing of these neurons decreased the persistence of navigation behavior, while sparse activation of these neurons could produce goal-directed running in a reproducible but arbitrary direction. These data suggest that this fan-shaped body representation of odor may be involved in setting a persistent goal direction during olfactory navigation, which would be required for surging behavior. However, other circuits are likely involved in the initial orientation toward odor, as well as in offset-evoked search behavior. While the function of most fan-shaped body local neurons and tangential neurons has not yet been determined, the connectivity patterns present in fan-shaped body local neurons suggest that they may be used in general for performing vector computations on the inputs encoded by columnar neurons (Hulse et al. 2021). A second group of local neurons, h∆B, were recently shown to compute an allocentric heading-direction signal (Lyu et al. 2022; Lu et al. 2022), suggesting that one function of fan-shaped body local neurons may be to perform computations in allocentric space. Finally, another group of local/output neurons called FC1 were recently shown to encode a goal direction in the context of orientation relative to a visual target (menotaxis, Pires et al. 2022). Thus, a common function of local neurons may be to encode diverse goals in allocentric space.

Like the mushroom body, the fan-shaped body appears to have a much smaller number of outputs than inputs. A notable output tract from the fan-shaped body to the LAL is formed by the PFL3 neurons (Fig. 5b; Stone et al. 2017; Hulse et al. 2021; Rayshubskiy et al. 2020). Intriguingly, PFL3 neurons receive phase-shifted information from the compass system in the ellipsoid body, producing two representations of heading that are each shifted by ~ 90° in the two hemispheres of the brain. Numerous modeling studies of the FB have hypothesized that this connectivity pattern uniquely positions PFL3 neurons to steer insects toward goal directions (Stone et al. 2017; LeMoel et al. 2019; Hulse et al. 2021; Goulard et al. 2021; Sun et al. 2021; Matheson et al. 2022). In these models, PFL3 neurons compare a goal direction, represented as a localized bump of activity across the FB, with the current heading of the fly, represented in the compass. The two shifted representations of heading in PFL3 neurons allow them to compute whether a left or right turn would bring the fly in line with its goal heading. PFL3 neurons provide input to LAL descending neurons that have been shown to drive turning (see below). Thus, PFL3 neurons have been proposed to integrate goal direction information and drive turning to maintain a stable goal-directed heading (Fig. 5c; Matheson et al. 2022; Stone et al. 2017; Sun et al. 2021; Goulard et al. 2021; Le Moël et al. 2019). Recent experimental findings support this model, and provide additional detail on how representations in central complex are “read-out” to produce goal-directed steering (Pires et al. 2022; Westeinde et al. 2022). Given emerging evidence that the fan-shaped body play a role in setting navigational goals, this area may be a possible neural substrate of the various “surge” behaviors observed in arthropod taxa. The diverse sensory inputs to the fan-shaped body may allow for goal directions to be computed based on flow, comparisons between sensors, or spatial memory. Investigating how diverse goal directions are computed in the fan-shaped body will be a fruitful area for future research.

### Pre-motor and motor circuits

Directed locomotion for navigation requires descending input from the brain to the ventral nerve cord (Fig. 4c). In the fly, there are around 350 descending neurons (DNs) per hemisphere, about 100 of which are now genetically accessible (Namiki et al 2018). A major question in descending motor control is whether motor actions are evoked by a small number of “command-like neurons”, or whether they are governed by population activity. Current data provide evidence for both models. Some behaviors, such as backwards walking, can be evoked by a single descending neuron, called “moonwalker” that is both necessary and sufficient for this behavior (Bidaye et al. 2014). In contrast, control of walking appears to be distributed across a population of DNs. At least three DNs in flies have been directly implicated in control of walking and turning: DNa01, DNa02, and DNp09 (Chen et al. 2018; Rayshubskiy et al. 2020; Bidaye et al. 2020). However, a recent imaging study of all DNs outside the SEZ found that ~ 60% participated in walking (Aymanns et al. 2022), consistent with a population code for control of walking and turning. Flight maneuvers also appear to be encoded at a population level. A population of 30 DNs were recently described that modulate wing beat amplitude and frequency to cause turns or straight trajectories of various speeds (Namiki et al. 2022).

In addition to controlling forward velocity and turning, the LAL may also play a role in generating more complex patterns of locomotor behavior. In moths, both descending and contralateral projection neurons, have been shown to respond to pheromone (Kanzaki et al. 1991; Namiki et al. 2014). Several of these neurons exhibit striking “flip-flop” activity, in which odor causes the neurons to switch their activity from high-firing rate to low or vice-versa (Olberg 1983; Kanzaki et al. 1994; Kanzaki and Mishima 1996; Namiki et al. 2014). Flip-flop activity in some descending neurons is correlated with zig-zagging head-movements (Kanzaki and Mishimi 1996) which are also observed during odor-offset-evoked casting behavior (Kanzaki et al. 1992). The LAL receives direct olfactory input from the mushroom body (Rayshubskiy et al. 2020; Li et al 2020; Scaplen et al. 2021) and from the superior protocerebrum (Namiki et al 2014). Thus, one hypothesis is that the casting or search component of olfactory navigation may be generated at the level of the LAL based on direct input from the mushroom body/lateral horn, rather than in the central complex. Consistent with this idea, studies in flies observed offset search when stimulating olfactory neurons in the mushroom body and lateral horn, but not when stimulating at the level of the fan-shaped body (Matheson et al. 2022).

Descending neurons can also control stopping—a behavior associated with gathering additional information during olfactory search (Demir et al. 2020). In flies, a group of neurons known as Pair 1 evoke stopping (Lee et al. 2021), although it is not known whether these neurons receive olfactory input. Another DN in larvae, known as PDM-DN, has been shown to evoke stop-turns and is required for proper olfactory navigation (Tastekin et al. 2018). Thus, DNs may be one locus where pausing behavior is initiated to obtain additional information about the odor environment. However, more experimental work will be required to understand how these DNs fit into complete sensory-motor circuits.

### Evolution and development of neural circuits across arthropods

Despite over 600 million years of evolution, the basic structures of the arthropod brain appear to be highly conserved (Loesel et al. 2013). Olfactory lobes that receive input from the olfactory receptor neurons in the first antenna are a common feature of arthropod brains (Schmidt and Mellon 2010; Sombke et al. 2011), despite immense variations in the size and morphology of the antennae themselves (Elgar et al. 2018; Derby 2021). These are generally divided into glomeruli and innervated by both local interneurons and projection neurons (Loesel et al. 2013). Mushroom bodies are found across insects (Strausfeld et al. 2009), although the inputs to this structure can differ across species. The mushroom body receives primarily olfactory input in some species (Li et al. 2020), and primarily visual input in others (Lin and Strausfeld 2012) and can persist in the absence of olfactory input (Strausfeld et al. 2009). In crustaceans, a structure known as the hemi-ellipsoid body receives input from the olfactory lobes (Wolff et al. 2017), and has recently been suggested to be homologous to the mushroom body, as they share many structural and molecular features (Wolff et al. 2015, 2017; Strausfeld et al. 2020).

The central complex (or “central body”) has been observed in diverse arthropod lineages, including insects, crustaceans, chelicerates (spiders, scorpions, horseshoe crabs), onychophorans (velvet worms), and chilopods (centipedes) (Homberg 2008; Loesel et al. 2013; Thoen et al. 2017; Chou et al. 2022). While the gross morphology of this structure differs across clades, many of its basic cell types and connectivity motifs appear to be well-conserved at least between flies and bees (Hulse et al. 2021; Sayre et al. 2021). Functional components of the central complex are structurally conserved across arthropods, although with morphological differences. The ellipsoid body forms a separate donut-shaped structure in flies, while in other insects, and crustaceans, it forms the lower division of the fan-shaped central body (Thoen et al. 2017; Sayre et al. 2021; Chou et al. 2022). The ellipsoid body and the lower division of the central body share strong GABA immunoreactivity (Homberg et al. 1999), likely arising from the ring neurons that carry sensory input to this structure (Heinze and Homberg 2008; el Jundi et al. 2014; Omoto et al. 2017; Fisher et al. 2019; Kim et al. 2019).

A lateral accessory lobe or lateral complex is also found in many insect species (Namiki and Kanzaki 2016). Descending neurons originating in the LAL have been shown to influence steering in both moths (Kanzaki and Mishima 1996) and flies (Chen et al. 2018; Rayshubskiiy et al. 2020). The LAL receives ascending input from the ventral nerve cord in both locusts (Homberg 1994) and crickets (Zorovic and Hedwig 2011). LAL-like structures have been observed in several crustaceans species, including stomatopods, crayfish, water fleas, and isopods (Thoen et al. 2017; Utting et al. 2000; Kress et al. 2016; Kenning and Harzsch 2013).

Both the conservation and the evolutionary variation observed across arthropod brain structures must ultimately arise from developmental processes. Significant changes in central brain structures occur between the larval and adult forms of *Drosophila*. For example, in larvae, the antennal lobe has a similar structure but many fewer glomeruli and receptors (Berck et al. 2016; Bates et al. 2020), the mushroom body is simplified (Truman et al. 2022), and the central complex is severely reduced or absent (Reibli et al. 2013; Andrade et al. 2019), though a LAL remains (Cardona et al. al 2009). Despite significant remodeling of the nervous system during metamorphosis (Truman et al. 2022), intriguing correspondences between larval and adult neurons have been observed. For example, the same neurons (moonwalker/mooncrawler) produce backward walking in adults (Bidaye et al. 2014), and backward crawling in larvae (Carreira-Rosario et al. 2018), despite striking differences in the physics and control of locomotion. Likewise, Pair 1 neurons produce stopping in both adults and larvae (Lee et al. 2021). These studies suggest that central control of high-level behaviors may be preserved even when the details of locomotion and body plan are completely different.

Conserved central brain structures, such as the mushroom body and central complex, arise from conserved stem cells, called neuroblasts, that proliferate to generate specific conserved cell types (Urbach and Technau 2004; Kunz et al. 2012; Yang et al. 2013; Yu et al. 2013; Walsh and Doe 2017). For example, different Kenyon cell types are generated sequentially from mushroom body neuroblasts (Lee and Luo 1999), and the columns of the central complex arise from four medial neuroblasts, called DM1-4, which decussate to form the characteristic scaffold of the fan-shaped body (Andrade et al. 2019; Sullivan et al. 2019). The generation of different cell types is controlled by the expression of a series of temporally restricted transcription factors and RNA binding proteins (Ren et al. 2017; Sullivan et al. 2019). Changes to the timing of these developmental programs results in the formation of a functional, but incomplete, central complex in the larval flour beetle (Koniszewski et al. 2016; Farnworth et al. 2020). Likewise, differences in the timing of mushroom body development relative to hatching vary across species (Farris and Sinakevitch 2003). Increases in both the number of neural progenitors, and the time over which these progenitors divide, contributes to the formation of some of the largest mushroom bodies among all insects in honeybees (Farris et al. 1999). Thus, changes to genes regulating neuroblast number, the timing of cell proliferation, and the expression of temporal transcription factors that specify cell identity, may drive the diversification of neural circuits to support behavior in diverse environments. Tools based on these genes may allow for experimental access to conserved cell types in diverse organisms (Farnworth et al. 2020), and for manipulation of circuit structures with consequences for behavior.

### Toward a comparative approach to olfactory navigation

In this review, we have tried to summarize our current state of knowledge about the diversity of olfactory navigation behaviors in arthropods and the potential circuit mechanisms underlying them. Several themes emerge from this review. First, despite differences in physiology across species and plume dynamics across environments, many arthropods rely on shared sensory strategies (flow detection, spatial comparison, short-term memory) to determine which way to go when searching for an odor source, and move with a conserved set of high-level behavioral features (surging, searching, pausing). Second, arthropods rarely rely on a single sensory cue or strategy to find an odor source, but rather appear to be able to integrate many sources of information coherently. Finally, the arthropod brain is built on a scaffold of conserved structures (antennal lobes, mushroom bodies, central complex), many of which have highly structured and conserved connectivity. How can the same brain scaffold be adapted to enable animals to effectively navigate toward odor goals in diverse environments, using diverse modes of locomotion? Given the ubiquity of olfactory navigation behavior in arthropods, its crucial role in animal survival and reproduction, and the conservation of underlying brain structures described above, we propose that addressing this question through a comparative study of the neural basis of olfactory navigation is likely to provide great insight into the organization and “functional logic” of neural circuit assembly.

How do species-specific differences in olfactory navigation arise? The most notable differences between species include which odors are innately attractive or aversive, what temporal patterns of odor fluctuation are required to drive movement upwind or upstream, and the characteristic frequencies and timescales of behavioral features such as surging, searching, and stopping. Thus far, the most progress has been made in understanding cross-species differences in odor preferences, which are mostly driven by changes in the repertoire of olfactory receptors and the structure of peripheral olfactory circuits. For example, specialization by *Drosophilia sechellia* on the acidic noni fruit has been linked to increased sensitivity to noni odor in a specific ORN, OR22a, an increased number of OR22a neurons, and enlargement of the corresponding glomerulus (Auer et al. 2020). More generally, the OR olfactory receptor family, which are unique to insects, have been shown to broadly respond to esters and alcohols (including hydrophobic or amphipathic esters and alcohols), while the IR family of olfactory receptors, which are expressed across arthropods, are selective for water-soluble acids and amines (Silbering et al. 2011). Differences in the selectivity of behavior for specific temporal patterns of odor stimulation have been linked to differences in the representation of odors, either with transient or persistent responses, at the level of the antennal lobe (Zocchi et al. 2022). However, whether central circuits for olfactory navigation differ across species, and how these circuit changes might be related to differences in behavior is not known. In *Drosophila* species, evolution of odor preference during courtship behavior has been linked to changes in central, rather than peripheral, odor selectivity (Seeholzer et al. 2018). Many central circuit modifications could underlie differences in the odor selectivity or dynamics of search behavior across organisms, including changes in neuron number, neuronal connectivity, synapse dynamics, and peptide and peptide receptor expression. A deeper understanding of the role of central neural circuits in olfactory navigation will be critical to make sense of how species-specific variations have emerged.

What tools and resources will be needed to more fully understand how the arthropod brain generates olfactory navigation behavior? A critical first step will be quantitative comparisons of search and navigation behavior across species in similar behavioral paradigms with well-controlled sensory stimuli. The explosion of tools for high throughput tracking and quantification of behavior using methods based on deep learning makes these previously challenging approaches newly possible (Mathis et al. 2018; Pereira et al. 2019). Complete connectomes for the fly brain and ventral nerve cord are in progress and will likely be made public soon (Dorkenwald et al. 2022; Phelps et al. 2021). Complementary work on the connectomes of other arthropods will allow us to understand which pathways are conserved and which vary across evolution. However, functional studies that ask how representations of odor, flow, locomotion, and environment change across each of the regions described here will also be required to understand how the arthropod brain effectively integrates different types of information to produce coherent goal-directed behavior. New tools for fast imaging across brain areas (Aimon et al. 2019), and for sparse imaging within developmentally defined populations (Isaacman-Beck et al. 2020), will be particularly useful in this regard. In addition, tools for labeling and manipulating conserved cell types across species will be needed. A promising avenue here are the newly developing tools to access cells based on developmental lineage and the expression of conserved transcription factors (Farnworth et al. 2020). Such tools would be more likely to generalize across species than current genetic drivers which are based on the expression of regulatory regions upstream of brain-expressed genes (Pfeiffer et al. 2008; Tirian and Dickson 2017). Moreover, they might allow for the manipulation of functionally related cohorts of neurons, and ultimately an understanding of how neural circuits evolve through changes in developmental pathways.

## Conclusions

Olfactory navigation is an ancient behavior that is critical for the survival and reproduction of most arthropod species. While the odors that drive attraction, the physical structure of odor plumes and trails, and the modes of locomotion differ greatly across arthropods, many of the core computations and behavioral motifs involved in getting to an odor source appear remarkably conserved. New tools for comparing the behavior, cell types, and organization of neural circuits across species have renewed interest in comparative approaches to understanding neural function. We propose that such comparative study of olfactory navigation in arthropods is likely to elucidate fundamental principles of neural circuit development, organization, and evolution.

## Data Availability

No new data were generated during this study.
